# Factors Influencing the Lives of Parents of Children With Autism Spectrum Disorder in Saudi Arabia: A Comprehensive Review

**DOI:** 10.7759/cureus.48325

**Published:** 2023-11-05

**Authors:** Shahad M Alkhonezan, Manal M Alkhonezan, Yara Alshayea, Hanan Bukhari, Rheem Almhizai

**Affiliations:** 1 Medicine and Surgery, Imam Mohammad Ibn Saud University, Riyadh, SAU; 2 Pediatric Medicine, Imam Mohammad Ibn Saud University, Riyadh, SAU

**Keywords:** parents' experiences, asd, autism spectrum disorder, mental health, children

## Abstract

Autism spectrum disorder (ASD) is a common neurodevelopmental disorder globally, presenting with core deficits in social interaction, repetitive behaviors, and communication abilities. This systematic review examined the factors influencing parents' experiences raising children with ASD in Saudi Arabia. Derived from a pool of 14 studies, eight were selected based on their focus on the impact of autism on family life. Results showed that mothers of children with ASD frequently reported heightened anxiety, self-stigmatization, and diminished quality of life. The quality of the parents' marital relationship and the caregiver's sex played crucial roles in determining emotional and behavioral outcomes in children with ASD. Additionally, societal reactions, feelings of embarrassment within families, and access to resources significantly influenced caregivers' experiences. Financial strains were common, with income inconsistencies affecting perceived quality of life. The severity of ASD and its duration also emerged as pivotal factors affecting families. Furthermore, the coronavirus disease 2019 pandemic intensified stress levels among parents, particularly mothers, emphasizing the need for psychological support. Our review findings highlight the importance of enhanced social support and recognition of stressed parents with autistic children in healthcare settings.

## Introduction and background

Autism spectrum disorder (ASD) is among the most prevalent neurodevelopmental disorders globally [1]. According to the Diagnostic and Statistical Manual of Mental Disorders, Fifth Edition (DSM-5), ASD is characterized by persistent deficits in social communication and interaction, as well as restricted, repetitive patterns of behavior, interests, or activities. The manifestations of these deficits can vary in range and severity and often evolve as children develop new skills [1]. Globally, one in 160 children is diagnosed with autism, which typically first appears in childhood [1]. This research also highlighted that changes in routines can particularly challenge children with ASD, potentially causing significant psychological strain on their parents [1]. Addressing the needs of these children can be daunting due to the condition's chronic nature, associated mental health comorbidities, the intensive interventions required, and challenges accessing support. Research on ASD in Saudi Arabia has expanded since 2008, yet it remains limited compared to Western countries. For instance, the prevalence rate of autism in developed countries ranges from 39 to 77 per 10,000. In contrast, a recent systematic review found that the prevalence of ASD in Arabian Gulf countries, including Saudi Arabia, ranged from 1.4 to 29 per 10,000, which is lower than the rates reported in developed nations [2].

Furthermore, the daily challenges of caring for these children can significantly affect the parent's mental health and their capacity to address the demands of their child and family [3]. In a study by Almansour involving 100 participants (50 parents of children with ASD and 50 parents of neurotypical children as a control group), parents with autistic children exhibited higher levels of depression and anxiety than the control group [4]. However, other research has shown that parents reporting higher satisfaction in their relationships experience less anxiety, positively influencing their child's emotional and behavioral outcomes [5]. Concerning income, Alshaigi et al. reported that a majority (85.7%) of caregivers felt their income was inadequate. Those with lower incomes were likelier to experience a reduced quality of life [6].

Conversely, three other studies found no significant relationship between income and quality of life for such families [7-9]. One Saudi study noted a decline in parental depression when a child with ASD demonstrated adaptive behavior. Parental anxiety was notably influenced by the child's emotional and behavioral challenges; however, this effect was less pronounced among parents who reported more satisfying marital relationships [5]. This review aims to identify the various factors influencing parents' experiences when raising a child with ASD in Saudi Arabia.

Methods

**Figure 1:**
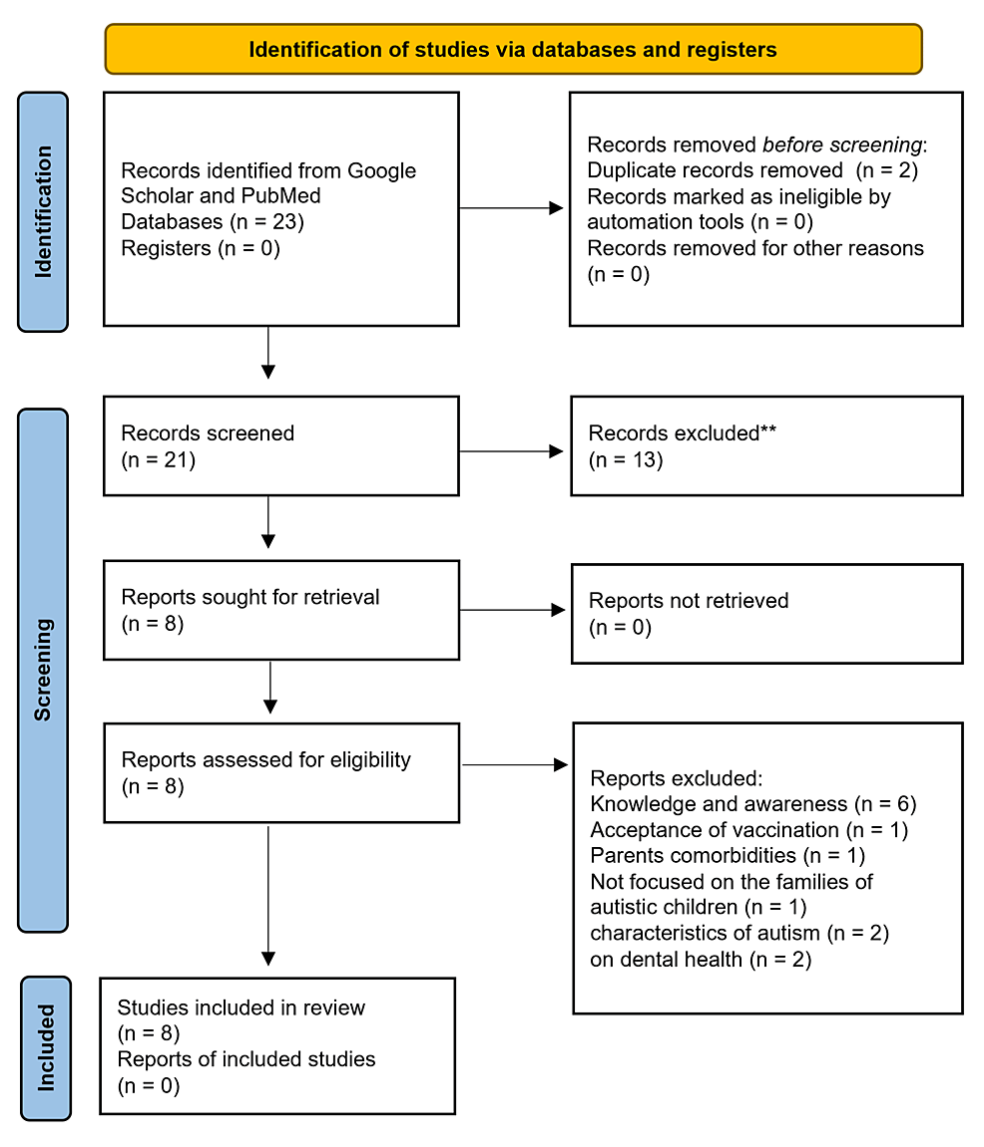
PRISMA Flowchart PRISMA, Preferred Reporting Items for Systematic Reviews and Meta-Analyses.

We conducted a comprehensive review of cross-sectional quantitative studies in Saudi Arabia examining the impact of autism on family life. We searched electronic databases, including PubMed and Google Scholar, using the keywords: "autism spectrum disorder" "Saudi Arabia," and "parents". Of the 21 identified studies, we included eight that exclusively addressed the effect of autism on family life. We excluded studies that did not focus on the impact of autism on the parents’ well-being, which were: six studies that were knowledge and awareness studies, one study on vaccine acceptance, one parents' comorbidities study, one family resident doctors' study (not on families of autistic children), two on the characteristics of autism, and two studies on dental health. From the selected studies, we extracted statistically significant factors (p-value < 0.05) (Figure 1).

## Review

From the studies reviewed, we identified factors significantly impacting the families of children with autism using the p-value. The p-value is a critical metric in research that indicates the probability of obtaining a specific result when the null hypothesis is true. A p-value below 0.05 typically suggests that the observed result is statistically robust, allowing researchers to gauge the validity of their findings (Table 1, Table 2) [10].

**Table 1:** Factors affecting caregivers of autistic children: a comparative analysis ASD, autism spectrum disorder; COVID-19, coronavirus disease 2019; QOL, quality of life; N/A, not applicable.

Author, Year	Caregiver Sex	Marital Relationship Impact	Impact on Work Life	Social Life Limitations
Khusaifan et al., 2022 [5]	N/A	Relationship satisfaction buffered the impact of children's issues on parental anxiety.	N/A	N/A
Alenazi et al., 2020 [6]	Female caregivers had a 3x lower QOL.	Not significant	72.6% reported less work accomplishment in the past 4 weeks due to emotional problems. Unemployed caregivers had a 2x lower QOL.	57.1% experienced interference with social activities due to their children’s issues.
Alshaigi et al., 2020 [7]	Mothers felt more self-stigmatized (41.75%) compared to fathers.	N/A	N/A	64.9% felt society doesn't understand children with ASD; 50.3% said ASD affected their vacations.
Alshahrani et al., 2021 [8]	N/A	N/A	N/A	Not significant
Khusaifan et al., 2021 [9]	N/A	N/A	N/A	Family and parental stress impacted life satisfaction levels.
Althiabi, 2021 [11]	Mothers showed higher anxiety during COVID-19.	N/A	N/A	N/A
Khan et al., 2020 [12]	N/A	N/A	N/A	37.7% felt their children caused family embarrassment; 63.9% were affected by societal reactions to their child.

**Table 2:** Influential factors on caregivers of children with ASD: an overview ASD, autism spectrum disorder; COVID-19, coronavirus disease 2019; QOL, quality of life; N/A, not applicable.

Author, Year	Effect of Income	Child's Sex	Birth Order	Disease Severity and Duration	Resource Availability	COVID-19 Impact
Alenazi et al., 2020 [6]	85.7% of caregivers with low income had a significantly lower QOL.	Parents of female children had a higher likelihood of poor QOL, but not statistically significant.	First birth order and disease duration (≥ 5 years) led to poorer parental QOL.	Same as 'Birth Order' column.	N/A	N/A
Alshahrani et al., 2021 [8]	Not significant	N/A	N/A	Only severe autism raised parental depression risk.	N/A	N/A
Khusaifan et al., 2021 [9]	Not significant	N/A	N/A	N/A	Resource availability moderated stress and life satisfaction via social support.	N/A
Althiabi, 2021 [11]	N/A	Higher anxiety when caring for female children.	N/A	N/A	41 sought help in the pandemic, highlighting a need for varied support, especially for mothers.	Significant rise in parental anxiety during COVID-19, especially for parents aged 26-35 years.
Khan et al., 2020 [12]	N/A	N/A	N/A	N/A	31.1% lacked nearby autism centers; 72.1% lacked private schools; 91.8% spent significantly on their child. 57.4% attended autism conferences; 72.1% felt a lack of Arabic ASD resources.	N/A
Alhuzimi, 2021 [13]	N/A	Child gender negatively impacted parental stress.	N/A	ASD symptom severity correlated with parental stress.	Frequency & usefulness of ASD support significantly affected parental stress and emotional well-being.	40.7% lost jobs due to COVID-19; 27.3% worked from home; 94% reported increased stress, 78.8% said their emotional well-being was affected by COVID-19.

Mothers of children with autism experienced elevated anxiety and self-stigmatization rates (41.75%) [7]. They also had a threefold increased likelihood of reporting a diminished quality of life [6] and heightened anxiety during the coronavirus disease 2019 (COVID-19) pandemic compared to fathers [11].

Parental satisfaction in marital relationships influenced their child's emotional and behavioral outcomes. Specifically, parents reporting higher marital satisfaction observed positive impacts on their child's emotions and behaviors [5]. Caregivers frequently noted disruptions in their social activities (57.1%) [6] and challenges arising from societal reactions to their autistic children (64.9%) [7] and 63.9% in another study [12], as well as feelings of embarrassment within their families (37.7%) [12].

Two studies indicated that caregivers of female autistic children experienced more stress and anxiety than male children [11,13]. However, one study reported that while parents of female autistic children were two to three times more likely to indicate a poorer quality of life than those of male autistic children, this difference was not statistically significant (p = 0.1) [6]. While income was not a determining factor in three studies [7-9], one study revealed that 85.7% of caregivers felt their income was insufficient, correlating low income with a decreased quality of life [6].

One study associated having a firstborn child with ASD and higher disease severity with a reduced quality of life [6]. Conversely, two other studies identified only disease severity as a significant factor [11,13]. The correlation between family stress and life satisfaction was notably diminished when considering social support [9]. Over 30% of families (31.1%) lacked proximity to an autism center, and most (72.1%) lived in areas without suitable private schools. A significant proportion (91.8%) of families incurred substantial expenses for their child's care.

While 45.9% of families deemed government facilities adequate, 57.4% participated in conferences to better understand autism. However, 72.1% believed there were insufficient Arabic resources on autism [12]. The frequency and efficacy of ASD support significantly swayed parental stress levels. Subsequent analyses revealed that the distress experienced by parents and the efficacy of ASD support, combined with the quality of parent-child interactions, were substantial determinants of parental stress. Positive impacts on emotional well-being were linked to both the regularity and efficiency of ASD support [13].

During the COVID-19 outbreak, 41 respondents sought assistance, and 135 parents detailed their mental health care needs. Findings highlighted a pronounced need for psychological support, primarily for mothers, in addition to financial aid, remote training, online counseling, guidance for managing behavioral issues at home, workshops, support groups, and dedicated child play areas [11].

The pandemic affected parents' employment: 40.7% were jobless, attributing their unemployment to the pandemic, while 27.3% transitioned to working from home. Most (94%) cited heightened stress levels due to the pandemic, and 78.8% felt their emotional wellbeing had deteriorated [13]. Notably, parental anxiety levels fluctuated between pre-pandemic times and during the COVID-19 crisis, with a marked rise during the pandemic. Increased anxiety levels were significantly more prevalent among parents aged 26-35 than other age groups [11].

Discussion

This comprehensive review was conducted to identify the factors that affect the lives of parents of autistic children in Saudi Arabia, which hopefully would guide future research into the causes of these factors. With the increase in awareness of ASD in the Arab world, Saudi Arabia had the highest number of research on ASD [14], but even with that the studies on parents of autistic children are few and on siblings of autistic children are currently non-existent. 

Sociocultural and Islamic traditions and values also have strong influences on shaping the family dynamics in Saudi families. It is culturally and religiously believed that fathers are the financial providers. Mothers, culturally, are expected to contribute to child-rearing and the house [15]; as the child-rearing load is on the mother that could be one of the causes of mothers of autistic children having a lower quality of life in Saudi Arabia.

Similar to findings in studies conducted in Saudi Arabia, a large number of studies conducted in the Arabian Gulf indicated that ASD has a major detrimental influence on the parents' lives. In Oman, mothers reported 3.4 times greater rates of depression, stress, and anxiety than fathers did when it came to their children with ASD [16]. Qatar also had lower quality of life (QOL) for parents, with mothers' QOL being noticeably worse [17]. A Kuwaiti study found that married mothers had lower rates of depression than single mothers, although mothers had greater rates of dysphoria and depression than fathers [18].

In reducing bias of the results in the studies reviewed, we relied on the p-value to identify the significant factors. The review's heavy reliance on the p-value as the sole determinant of significance could also be restrictive. Without considering effect sizes and confidence intervals, the magnitude and precision of observed effects might not be accurately represented. Additionally, the review does not account for potential biases inherent in the individual studies analyzed, which might skew the overall findings. It exclusively focuses on cross-sectional quantitative studies, potentially overlooking the depth and nuances of experiences that qualitative research might offer. By not having diverse study designs, the review may miss some contextual and culturally specific factors essential to understanding the challenges faced by families in Saudi Arabia. The geographical spread and demographic variation within Saudi Arabia, such as urban versus rural differences, are not explicitly addressed, which might further limit the generalizability of the conclusions drawn.

## Conclusions

Several studies in Saudi Arabia have identified multiple factors influencing children with autism and their parents' lives. These factors include the caregiver's sex, the quality of the parents' marital relationship, limitations in work and social lives, income implications, the child's sex, access to resources, adequate social support, the severity of the condition, and duration. Currently, there is limited national research on the effects of autism on families, especially as different cultures can introduce unique challenges and factors. In particular, the effects on families with female children diagnosed with ASD remain under-researched. It is imperative to conduct more studies to determine the underlying causes and subsequently offer the necessary support to these families. Healthcare providers should prioritize increasing social support, translating existing resources into Arabic, or creating new Arabic resources about autism to guide parents. Additionally, it's crucial to recognize and support parents, especially mothers, experiencing stress from raising a child with ASD. They are often more susceptible to stress and may have a diminished quality of life.
